# Monocyte subsets and monocyte-related chemokines in Takayasu arteritis

**DOI:** 10.1038/s41598-023-29369-3

**Published:** 2023-02-06

**Authors:** Mariana Freitas de Aguiar, Heron Torquato, Bruno Ramos Salu, Ana Cecília Diniz Oliveira, Maria Luiza Vilela Oliva, Edgar Julian Paredes-Gamero, Wayel H. Abdulahad, Elisabeth Brouwer, Alexandre W. S. de Souza

**Affiliations:** 1 Rheumatology Division, Department of Medicine, Universidade Federal de São Paulo–Escola Paulista de Medicina, Rua dos Otonis, 863, São Paulo, SP 04025-002 Brazil; 2 Faculdade de Farmácia, Centro Universitário Braz Cubas, Avenida Francisco Rodrigues Filho, 1233, Mogi das Cruzes, SP 08773-380 Brazil; 3 Department of Biochemistry, Universidade Federal de São Paulo–Escola Paulista de Medicina, Rua Três de Maio, 100. Fifth Floor, São Paulo, SP 04044-020 Brazil; 4 Faculdade de Farmácia, Tecnologia de Alimentos e Nutrição, Universidade Federal de Mato Grosso do Sul, Campo Grande, MS 79070-900 Brazil; 5 Department of Rheumatology and Clinical Immunology, University of Groningen, University Medical Center Groningen, Hanzeplein 1, 9713 GZ Groningen, The Netherlands; 6 Department of Pathology and Medical Biology, University of Groningen, University Medical Center Groningen, Hanzeplein 1, 9713 GZ Groningen, The Netherlands

**Keywords:** Immunology, Autoimmunity, Innate immune cells, Innate immunity

## Abstract

The pathogenesis of Takayasu arteritis (TAK) is poorly understood and no previous studies have analyzed monocytes in TAK. This study evaluated monocyte subsets and monocyte-related chemokines in the peripheral blood of TAK patients and healthy controls (HC). Monocyte subsets were identified as classical (CD14^+^CD16^−^), intermediate (CD14^+^CD16^dim^), and non-classical (CD14^dim^CD16^high^) in the peripheral blood. The chemokines CCL (C–C chemokine ligand)2, CCL3, CCL4, CCL5, CCL7, CXCL (C-X-C motif ligand)10, and CX3CL (C-X3-C motif ligand)1 were measured in the sera. Thirty-two TAK patients and 30 HC were evaluated. Intermediate monocytes were higher in TAK than HC [25.0 cells ×10^6^/L (16.7–52.0) vs. 17.2 cells ×10^6^/L (9.2–25.3); *p* = 0.014]. Active disease was associated with monocytosis (*p* = 0.004*)*, increased classical (*p* = 0.003), and intermediate (*p* < 0.001) subsets than HC. Prednisone reduced the percentage of non-classical monocytes (*p* = 0.011). TAK patients had lower CCL3 (*p* = 0.033) and CCL4 (*p* = 0.023) levels than HC, whereas CCL22 levels were higher in active TAK compared to the remission state (*p* = 0.008). Glucocorticoids were associated with lower CXCL10 levels (*p* = 0.012). In TAK, CCL4 correlated with total (Rho = 0.489; *p* = 0.005), classical and intermediate monocytes (Rho = 0.448; *p* = 0.010 and Rho = 0.412; *p* = 0.019). In conclusion, TAK is associated with altered counts of monocyte subsets in the peripheral blood compared to HC and CCL22 is the chemokine with the strongest association with active disease in TAK.

## Introduction

Takayasu arteritis (TAK) is a chronic granulomatous vasculitis that affects the aorta, its main branches, and pulmonary arteries^1^. The pathogenesis of TAK is still poorly understood; it involves primarily a cell-mediated immune response with a heterogeneous inflammatory infiltration in the vascular wall that comprises cluster of differentiation (CD)4^+^T cells, CD8^+^T cells, γδ T cells, B cells, natural killer cells, macrophages, multinucleated giant cells, and granulocytes^2,3^. In TAK, dendritic cells are co-localized with T cells around the *vasa vasorum* and in the adventitia of involved arteries^3,4^.

Activated T helper (Th)1 cells in TAK release tumor necrosis factor (TNF) and interferon-gamma that promote a persistent activation of monocytes and macrophages leading to granuloma formation^5^. Recent studies demonstrated a predominance of the Th1 and Th17 responses in the inflammatory cascade of the TAK pathogenesis^6,7^. Inflammatory abnormalities in arteries from TAK patients are possibly triggered by environmental agents such as infectious agents. One study showed distinct toll-like receptors (TLR) profiles in different arterial territories from patients with large-vessel vasculitis. The TLR1, TLR3, TLR5, TLR6, and TLR8 were expressed in some arteries, but TLR2 and TLR4 are ubiquitously present^8^. In addition, TAK patients present a specific blood microbiome profile in comparison with healthy individuals^9^.

Under inflammatory conditions, blood monocytes may migrate into affected tissues and differentiate into mononuclear phagocytic cells^10^. In addition to their ability to generate tissue macrophages and monocyte-derived dendritic cells, monocytes have a crucial role in phagocytosis and antigen presentation^11^. Three monocyte subsets can be distinguished by their phenotypic and functional characteristics depending on the surface expression of CD14 and CD16. The monocyte’s subsets include classical (CD14^+^CD16^−^), intermediate (CD14^+^CD16^dim^), and non-classical (CD14^dim^CD16^high^)^12^. The classical subset is the most abundant in the peripheral blood, this subset has a high phagocytic capacity, and it is the first subset recruited during acute inflammation. Intermediate monocytes are specialized in antigen presentation and produce large amounts of proinflammatory cytokines, such as TNF, interleukin (IL)-1β, and IL-6. Conversely, non-classical monocytes have an important role in endothelial patrolling and antiviral response^13–16^. The CD16^+^ monocytes are more differentiated cells compared to the classical monocyte subset^17^.

The monocytes' recruitment from the peripheral blood to inflammation sites is orchestrated by chemokines^18^. Classical monocytes express mainly the CC chemokine receptor type 2 (CCR2), while the intermediate and non-classical subsets express CX3C chemokine receptor type 1 (CX3CR1)^19^. The C–C chemokine ligand (CCL) 2 is the strongest and most selective ligand of CCR2, which can also bind to CCL7^20^. When monocytes differentiate into macrophages, CCR2 expression is lost, and cells express CCR1 and CCR5 which in turn have the chemokines CCL3, CCL4, and CCL5 as ligands^19,21^. These chemokines are crucial for monocyte migration and have been already implicated in the pathogenesis of inflammatory diseases, such as rheumatoid arthritis and atherosclerosis^22^. On the other hand, the CX3CR1-CX3CL1 axis is important for CD16^+^ monocyte’s kinetics and is related to the non-classical monocytes “patrolling behavior”^18,23^.

To date, no studies have analyzed peripheral blood monocytes in TAK as previous studies focused mainly on macrophage subsets and other inflammatory cells in the arterial wall infiltration in TAK^24,25^. The few studies approaching the profile of chemokines in TAK have shown discordant results^7,26–29^. No analyses regarding the interaction between chemokines and the distribution of a specific cell type were performed. Therefore, this study aims to evaluate the distribution of monocyte subsets in the peripheral blood and the serum monocyte-related chemokines’ profile in TAK, as well as analyze associations between monocyte subsets and serum chemokines regarding disease status, disease activity, and therapy.

## Results

### TAK patients and healthy controls

Thirty-two consecutive TAK patients and 30 healthy controls (HC) with similar mean age (45.6 ± 13.7 years vs. 44.1 ± 10.8 years, *p* = 0.651) and female/male ratio (30/32 vs. 28/30, *p* = 0.947) were included in the study. Table 1 describes the disease features and therapy of TAK patients. Twenty-seven (84.4%) patients received therapy for TAK, including immunosuppressive agents or biological agents at the inclusion. The TNFα inhibitors and tocilizumab were the biological agents prescribed (Table 1). Twelve TAK patients (37.5%) used prednisone with a median daily dose of 6.3 mg (2.5–20.0).

**Table 1:** Disease features and therapy for Takayasu arteritis.

Variables	Results (n = 32)
TAK features	
Median time since TAK diagnosis, months	150.0 (60.0–228.0)
Active disease, n (%)	8 (25.0)
Median ITAS2010	3.0 (2.0–4.0)
ESR, mm/hour	25.0 (6.0–38.0)
CRP, mg/dL	1.52 (0.66–6.30)
Angiographic types	
Type I, n (%)	5 (15.6)
Type IIa, n (%)	1 (3.1)
Type IIb, n (%)	2 (6.3)
Type III, n (%)	1 (3.1)
Type IV, n (%)	2 (6.3)
Type V, n (%)	21 (65.6)
Therapy for TAK	
Immunosuppressive agents, n (%)	21 (65.6)
Methotrexate, n (%)	12 (57.1)
Leflunomide, n (%)	7 (33.3)
Azathioprine, n (%)	2 (9.5)
Biologic therapy, n (%)	10 (31.3)
Adalimumab, n (%)	5 (50.0)
Tocilizumab, n (%)	4 (40.0)
Infliximab, n (%)	1 (10.0)
No therapy, n (%)	5 (15.6)

*CRP* C-reactive protein, *ESR* erythrocyte sedimentation rate, *ITAS2010* Indian Takayasu Clinical Activity Score, *n* number of patients, *TAK* Takayasu arteritis. The percentage of individual therapeutic agents refers to the whole number of patients using immunosuppressive and biological agents, respectively.

### Monocyte subsets in TAK patients and HC

**Figure 1:**
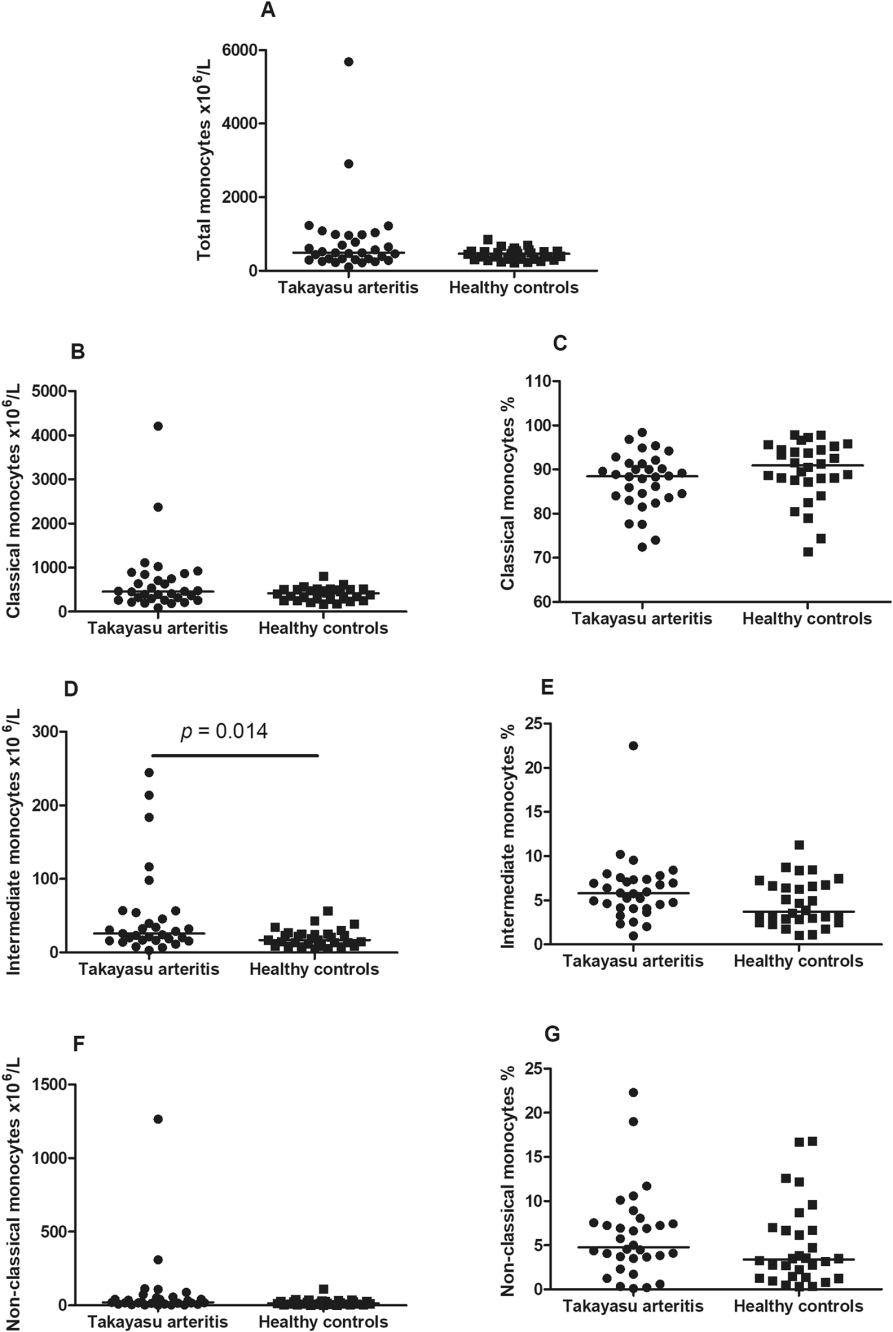
Monocyte subsets in the peripheral blood from patients with Takayasu arteritis and healthy controls. Patients with TAK present significantly higher number of intermediate monocytes than HC (**D**). However, TAK patients and healthy controls present similar number of total (**A**), classical (**B**) and non-classical monocytes (**F**). The percentage of classical (**C**), intermediate (**E**) and non-classical monocytes (**G**) were similar between TAK patients and HC. *HC* healthy controls, *TAK* Takayasu arteritis.

Patients with TAK presented a higher number of circulating intermediate monocytes compared to HC [25.0 cells ×10^6^/L (16.7–52.0) vs. 17.2 cells ×10^6^/L (9.2–25.3); *p* = 0.014]. However, no significant differences were observed between TAK patients and HC regarding total monocytes [493.1 cells ×10^6^/L (313.5–976.7) vs. 461.3 cells ×10^6^/L (314.0–544.1); *p* = 0.185], classical monocytes [453.1 cells ×10^6^/L (269.5–819.7) vs. 420.7 cells ×10^6^/L (278.0–506.4); *p* = 0.200] and non-classical monocytes [20.8 cells ×10^6^/L (9.7–49.3) vs. 14.2 (6.5–28.3); *p* = 0.125] (Fig. 1). In addition, no differences were observed between TAK patients and HC for the percentage of the classical [88.5% (83.7–91.4) vs. 90.9% (87.5–94.7); *p* = 0.111], intermediate [5.8% (4.1–7.4) vs. 3.7% (2.8–6.7); *p* = 0.066], and non-classical subsets [4.8% (3.5–7.5) vs. 3.4% (1.4–6.8); *p* = 0.149] (Fig. 1).

### Monocyte subsets and disease activity in TAK

**Figure 2:**
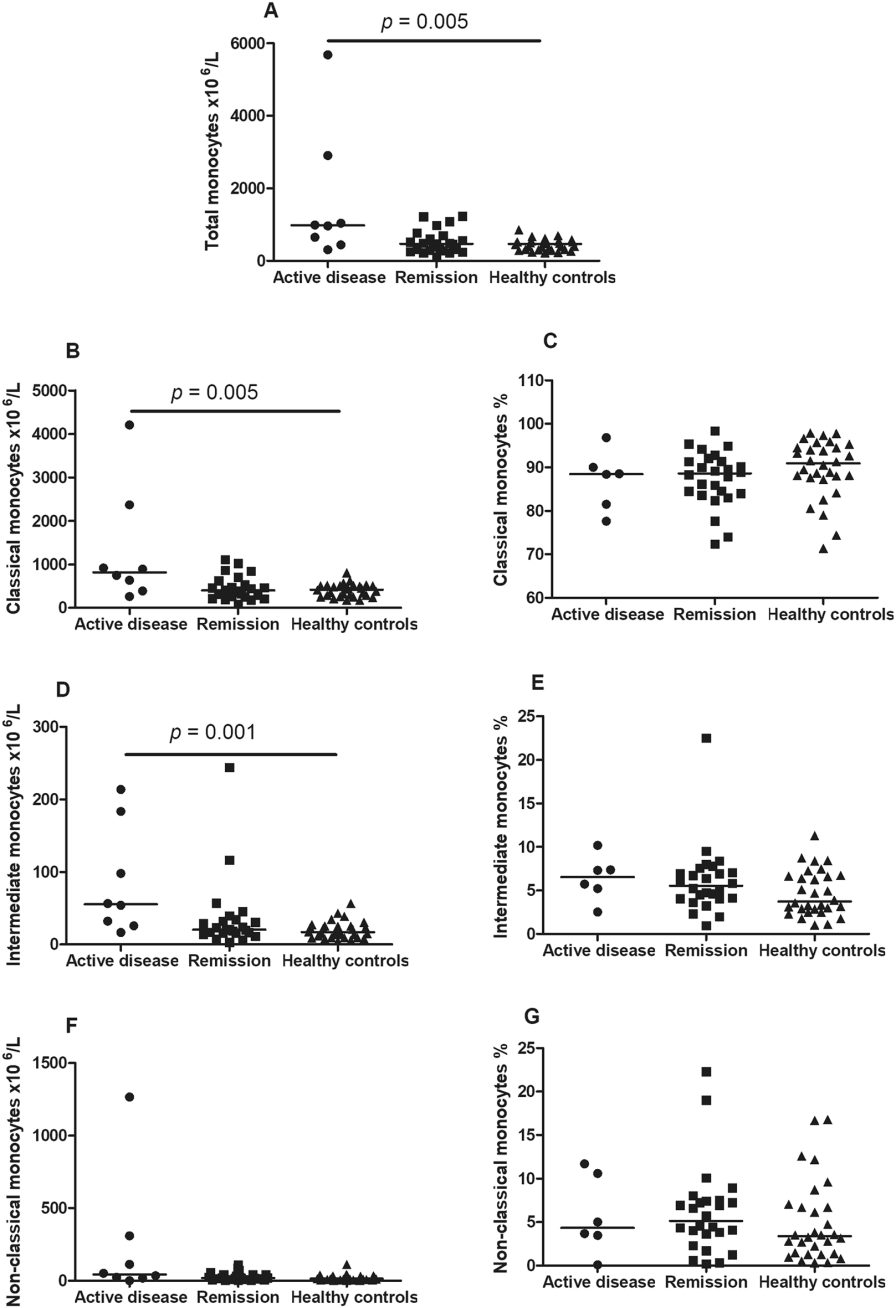
Monocyte subsets in the peripheral blood from Takayasu arteritis patients with active disease, and in remission versus healthy controls. Patients with TAK with active disease had significantly higher number of total monocytes (**A**), classical monocytes (**B**), and intermediate monocytes (**D**) than HC. No significant differences were observed regarding the number of non-classical monocytes (**F**) and the percentage of classical (**C**), intermediate (**E**) and non-classical (**G**) monocytes among TAK patients with active disease, remission and HC. *HC* healthy controls, *TAK* Takaysu arteritis. *The cutoff *p*-value was *p* < 0.016 for significant differences in the post hoc analyses according to Bonferroni’s correction*.*

The comparisons among active TAK (n = 8/32), TAK in remission (n = 24/32), and HC (n = 30) yielded significant differences regarding the number of total monocytes [976.1 cells ×10^6^/L (495.0–2438.4) vs. 465.8 cells ×10^6^/L (283.4–677.0) vs. 461.3 cells ×10^6^/L (314.0–544.1); *p* = 0.025], classical monocytes [818.8 cells ×10^6^/L (451.2–2004.9) vs. 404.0 cells ×10^6^/L (257.4–604.0) vs. 420.6 cells ×10^6^/L (278.0–506.4); *p* = 0.022] and intermediate monocytes [55.4 cells ×10^6^/L (27.5–162.2) vs. 20.7 cells ×10^6^/L (15.7–33.8) vs. 17.2 cells ×10^6^/L (9.2–25.3); *p* = 0.003]. However, no differences were found between active TAK, TAK in remission, and HC regarding the number of non-classical monocytes [43.3 cells ×10^6^/L (18.0–259.9) vs. 18.3 cells ×10^6^/L (8.4–40.3) vs. 14.2 cells ×10^6^/L (6.5–28.3); *p* = 0.089] in the peripheral blood, respectively. The percentage of the classical [86.2% (78.6–89.6) vs. 89.1% (84.5–91.9) vs. 90.9% (87.5–94.7); *p* = 0.171], intermediate [6.5% (3.7–8.1) vs. 5.5% (4.1–7.0) vs. 3.7% (2.8–6.7); *p* = 0.154], and non-classical subsets [6.3% (3.5–11.4) vs. 5.5% (2.6–7.2) vs. 3.7% (2.8–6.7); *p* = 0.265] was not different among these three groups (Fig. 2).

In the post hoc analyses, the main differences regarding the number of monocytes in the peripheral blood were found between TAK patients with active disease and HC for total monocytes (*p* = 0.005), classical monocytes (*p* = 0.005), and intermediate monocytes (*p* = 0.001). However, differences between TAK patients with active disease and those in remission were not significant according to Bonferroni’s correction (i.e., *p* < 0.016) for total monocytes (*p* = 0.029), classical (*p* = 0.023), and intermediate monocytes (*p* = 0.019) in the peripheral blood. In addition, TAK patients with active disease and those in remission had similar numbers of total monocytes and their subsets (*p* > 0.05) (Fig. 2).

### Longitudinal analysis of monocytes in TAK

Four TAK patients with active disease were reassessed for monocytes subsets in the peripheral blood when achieving remission at a median of 14.5 months (11.3–18.5) after the first assessment. Despite the low number of patients assessed longitudinally, an apparent decrease in the median number of cells in the peripheral blood was observed between active disease and remission in TAK in paired analyses of total monocytes [1972.0 cells ×10^6^/L (491.0–4986.0) vs. 411.0 cells ×10^6^/L (334.7–545.0)], classical monocytes [1,643.0 cells ×10^6^/L (423.9–3743.0) vs. 360.9 cells ×10^6^/L (271.4–462.6)], intermediate monocytes [118.9 cells ×10^6^/L (33.0–206.3) vs. 20.7 cells ×10^6^/L (14.6–22.2)], and non-classical monocytes [180.5 cells ×10^6^/L (30.5–1,026.0) vs. 40.6 cells ×10^6^/L (25.0–61.4)] from active disease to remission, respectively.

### Serum chemokines in TAK patients and HC

**Figure 3:**
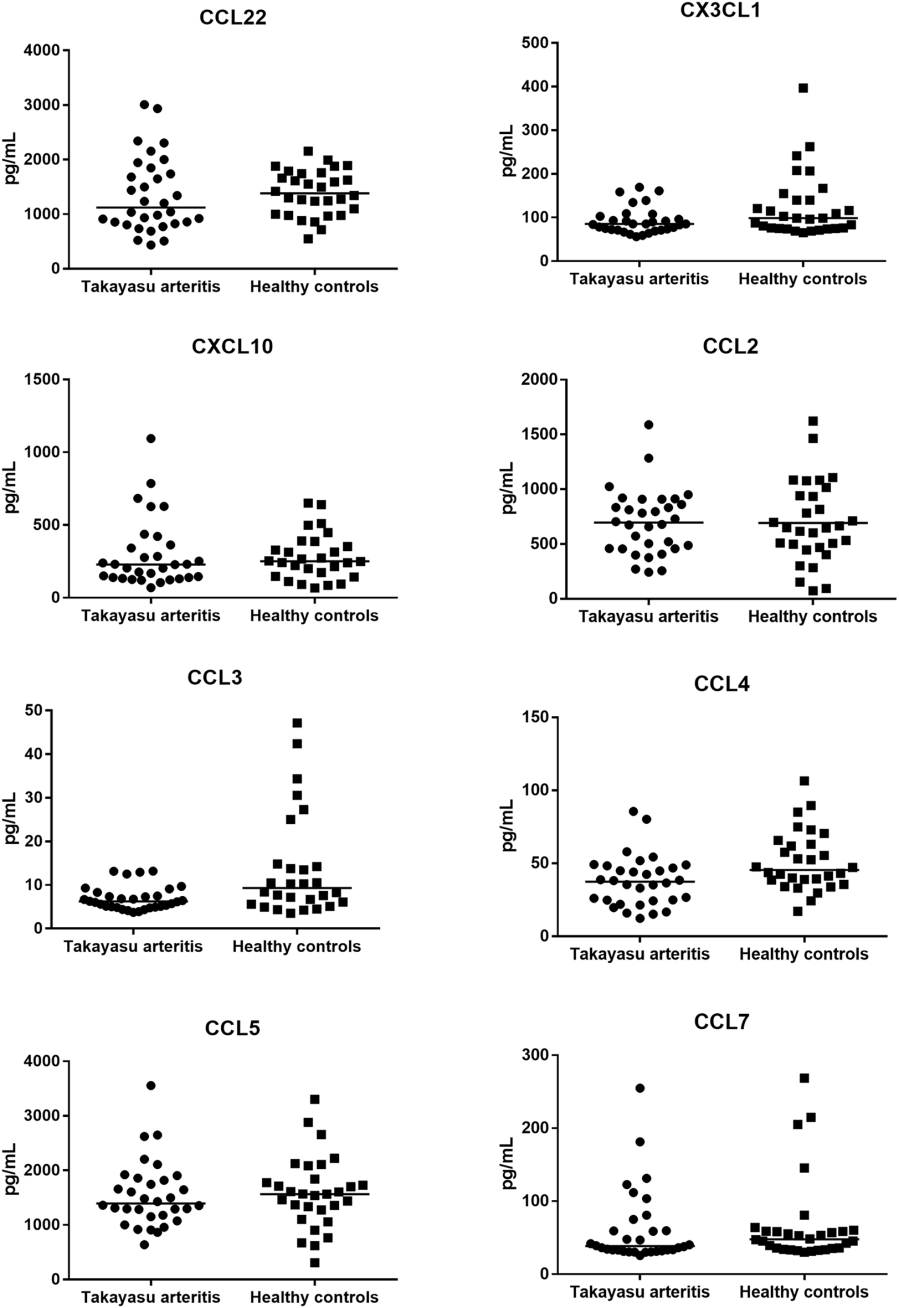
Serum chemokine levels in Takayasu arteritis patients versus healthy controls. Patients with TAK and HC presented similar serum chemokine levels, except for CCL3 and CCL4 levels which were higher in TAK patients than in HC. *HC* healthy controls, *TAK* Takayasu arteritis.

No significant results were observed between TAK patients and HC regarding serum levels of CCL22, CX3CL1, CXCL10, CCL2, CCL5, and CCL7 levels. However, TAK patients presented lower CCL3 [6.2 pg/mL (4.9–8.1) vs. 9.3 pg/mL (5.7–14.7); *p* = 0.009] and CCL4 [37.4 pg/mL (24.5–47.9) vs. 45.4 pg/mL (37.7–63.7); *p* = 0.008] compared to controls (Fig. 3) (Supplementary Table S1).

When subgroup analyses were performed comparing TAK patients with active disease (n = 5/32), TAK patients in remission (n = 27/32), and HC (n = 30), median serum CCL22 levels were significantly higher in patients presenting active disease (2030.5 ± 982.5 pg/mL) than patients in remission (1222.3 ± 552.6 pg/mL) and HC (1390.6 ± 410.9 pg/mL) *p* = 0.008 and *p* = 0.024, respectively. Conversely, median serum CCL3, and CCL4 levels were lower in patients in remission than in HC (*p* = 0.010 and *p* = 0.028).

### Correlations between monocyte subsets and serum chemokines with Indian Takayasu Clinical Activity Score 2010 and acute phase reactants in TAK

No correlations were found between total monocytes and monocyte subsets counts in the peripheral blood with erythrocyte sedimentation rate (ESR), serum C-reactive protein (CRP), or Indian Takayasu Clinical Activity Score (ITAS2010) score (Supplementary Table S2). The analysis of correlations between serum chemokine levels and acute phase reactants or ITAS2010 score yielded only a significant negative correlation between serum CCL2 levels and ITAS2010 score (rho = − 0.611; *p* = 0.046). No significant correlations were observed between serum levels of other chemokines and ESR, serum CRP or ITAS2010 score (Supplementary Table S3).

### Interactions between monocyte subsets and chemokines in TAK patients

Serum CCL4 levels were significantly correlated with the total number of monocytes (Rho: 0.489; *p* = 0.005), as well as, with classical and intermediate monocytes (Rho: 0.448; *p* = 0.010 and Rho: 0.412; *p* = 0.019, respectively). CCL7 was also significantly correlated with the number of non-classical monocytes in the peripheral blood (Rho: 0.360; *p* = 0.043). No other correlations were found between other serum chemokine levels and total monocytes or monocyte subsets in the peripheral blood of TAK patients (Supplementary Table S4).

### The impact of therapy on monocyte subsets and serum chemokines

Firstly, we analyzed the effect of prednisone use and its daily dose on monocyte subsets and circulating chemokines. TAK patients using prednisone dose ≥ 5 mg were compared to those on prednisone < 5 mg/day or no prednisone. Daily prednisone ≥ 5 mg was associated with a lower percentage of non-classical monocytes compared to daily prednisone < 5 mg [3.7% (1.3–4.1) vs. 6.9% (3.9–8.5), *p* = 0.011]. No other significant differences were observed in the absolute numbers and percentages of monocyte subsets in the peripheral blood regarding prednisone use in TAK patients. When the relations between prednisone use and serum chemokines were analyzed, TAK patients on a prednisone daily dose ≥ 5 mg presented lower serum CXCL10 levels than those using < 5 mg/day. No differences were observed regarding prednisone use and serum levels of other chemokines in TAK patients (Table 2). The prednisone daily dose also had a strong negative correlation with the percentage of non-classical monocytes (rho: -0,796; p = 0.002). (Supplementary Table S5). Correlations between daily prednisone dose and serum chemokines yielded no significant results (Supplementary Table S5).

**Table 2:** Impact of prednisone use on monocyte subsets and chemokines in TAK patients.

Variables	Prednisone ≥ 5 mg/day (n = 7)	Prednisone < 5 mg/day or no prednisone (n = 25)	*p*
Total monocytes, cells ×10^6^/L	523.1 (441.7–1085.2)	471.1 (286.1–972.7)	0.327
Classical monocytes, cells ×10^6^/L	472.5 (390.3–843.4)	412.2 (257.9–807.3)	0.305
Intermediate monocytes, cells ×10^6^/L	32.1 (20.1–57.1)	23.3 (16.2–49.9)	0.399
Non-classical monocytes, cells ×10^6^/L	16.3 (4.1–31.8)	22.6 (14.3–62.4)	0.245
Classical monocytes, %	89.60 ± 5.8	86.7 ± 6.3	0.293
Intermediate monocytes, %	4.6 (4.1–7.3)	5.9 (4.3–7.5)	0.569
Non-classical monocytes, %	3.7 (1.3–4.1)	6.9 (3.9–8.5)	0.011*
CX3CL1, pg/mL	85.2 (82.9–96.2)	81.8 (71.2–106.5)	0.741
CCL22, pg/mL	1035.0 (688.1–1497.0)	1201.0 (839.8–1896.5)	0.509
CXCL10, pg/mL	138.9 (104.7–203.6)	245.9 (154.5–433.2)	0.012*
CCL2, pg/mL	783.3 (459.4–831.8)	679.3 (456.7–911.0)	0.837
CCL3, pg/mL	7.5 (6.7–9.3)	5.7 (4.9–7.3)	0.116
CCL4, pg/mL	36.4 ± 13.8	38.0 ± 18.4	0.830
CCL5, pg/mL	1657.0 (1002.0–2206.0)	1360.0 (1230.5–1838.0)	0.767
CCL7, pg/mL	34.4 (33.4–59.3)	39.1 (31.4–78.1)	0.909

Results are presented as median and interquartile range or as a mean and standard deviation; n—number of patients; *—flags significant results.

The impact of immunosuppressive and biological agents on monocyte subsets and serum chemokine levels was also analyzed in TAK patients. Despite the lower number of total monocytes and classical monocytes in the peripheral blood in TAK patients on immunosuppressive agents, the comparison with those on biological agents and no therapy in the post hoc analyses yielded no significant results according to Bonferroni’s correction (i.e., *p* > 0.016) (Table 3). No other significant differences were observed for monocyte subsets and serum chemokine levels regarding therapy for TAK (Table 3).

**Table 3:** Impact of immunosuppressive and biologic therapy on monocyte subsets and chemokines in TAK patients.

Variables	Immunosuppressive agents (n = 17)	Biological agents (n = 10)	No therapy (n = 5)	*p*
Total monocytes, cells ×10^6^/L	334.8 (251.9–612.3)	654.7 (428.8–1225.6)	980.8 (387.7–3333.9)	0.039*
Classical monocytes, cells ×10^6^/L	324.4 (213.1–553.1)	581.4 (382.6–1044.4)	866.0 (357.9–2545.7)	0.023*
Intermediate monocytes, cells ×10^6^/L	21.4 (16.2–31.3)	33.4 (18.6–71.9)	39.7 (14.1–120.1)	0.361
Non-classical monocytes, cells ×10^6^/L	17.7 (5.3–27.5)	40.2 (13.3–63.4)	34.5 (14.5–669.1)	0.231
Classical monocytes, %	85.9 (82.7–90.8)	89.1 (86.8–92.6)	90.0 (81.2–92.1)	0.558
Intermediate monocytes, %	6.9 (4.9–7.9)	4.8 (4.0–7.3)	4.1 (2.8–5.8)	0.086
Non-classical monocytes, %	6.9 (2.1–8.5)	4.7 (3.2–6.8)	4.6 (2.9–1499)	0.875
CX3CL1, pg/mL	84.1 (70.0–99.4)	84.1 (70.3–103.3)	99.8 (78.2–108.5)	0.578
CCL22, pg/mL	1199.8 ± 493.6	1645.8 ± 904.5	1259.8 ± 706.5	0.258
CXCL10, pg/mL	215.9 (146.5–319.4)	207.9 (124.4–425.8)	231.2 (145.3–455.5)	0.897
CCL2, pg/mL	521.4 (388.2–917.1)	756.1 (614.4–852.3)	647.2 (514.3–860.2)	0.658
CCL3, pg/mL	5.7 (5.0–7.1)	6.6 (4.7–10.2)	7.3 (5.6–10.2)	0.410
CCL4, pg/mL	33.0 (23.1–48.4)	44.4 (23.6–48.5)	38.8 (23.8–64.0)	0.727
CCL5, pg/mL	1360.0 (1234.5–1701.5)	1417.5 (981.4–2132.5)	1858.0 (1075.0–2262.5)	0.862
CCL7, pg/mL	41.8 (31.1–92.3)	34.9 (31.9–50.6)	40.5 (35.1–151.0)	0.595

Results are presented as mean and standard deviation or as median and interquartile range. * Flags significant results; n—number of patients. Post hoc analysis for total monocytes: immunosuppressive agents vs. biological agents (*p* = 0.024), immunosuppressive agents vs. no therapy (*p* = 0.078) and biological agents vs. no therapy (*p* = 0.903). Post hoc analysis for classical monocytes: immunosuppressive agents vs. biological agents (*p* = 0.018), immunosuppressive agents vs. no therapy (*p* = 0.046) and biological agents vs. no therapy (*p* = 1.000). Bonferroni’s correction for post hoc analysis (*p* = 0.016).

## Discussion

In this study, we observed that TAK patients present an increased number of intermediate monocytes in the peripheral blood than HC, and disease activity in TAK was associated with an increased number of total monocytes, as well as classical and intermediate monocytes compared to HC. CCL22 was the only chemokine related to disease activity in TAK patients, whereas serum CCL3 and CCL4 were lower in TAK patients than in HC. CCL4 levels correlated with total monocytes and the classical and intermediate subsets in all TAK patients regardless of disease activity. Prednisone therapy was associated with lower serum CXCL10 levels and a lower percentage of non-classical monocytes in the peripheral blood of TAK patients. However, other therapies for TAK did not impact chemokines’ concentrations or monocyte subsets in the peripheral blood.

The expansion of the classical subset seen in active disease probably reflects the acute efflux of these monocytes from bone marrow to peripheral blood in response to an unknown trigger^19^. CD14^+^ CD16^−^ human monocytes express higher levels of chemokine receptors, such as CCR1, CCR2, CX chemokine receptor (CXCR)1, and CXCR2 which highlights their potential to migrate to inflamed tissues^30^. During the maturation of the human monocyte, mathematical modeling suggests that the classical subset leaves the bone marrow, and later in the bloodstream, classical monocytes differentiate first into intermediate and then into non-classical subset^31^.

Monocyte subsets in the peripheral blood were also analyzed in other forms of systemic vasculitis such as an antineutrophil cytoplasmic antibody (ANCA)-associated vasculitis (AAV), including granulomatosis with polyangiitis and microscopic polyangiitis, giant cell arteritis (GCA) and Behçet´s disease (BD)^32–35^.

The expansion of the intermediate subset of monocytes was also seen in the peripheral blood of BD patients, a multisystemic inflammatory syndrome that also has a strong role of the innate immunity in its pathogenesis^32,35^. This monocyte subset also seems to play a role in the pathophysiology of AAV since patients with active disease and those in remission presented a higher number of intermediate monocytes in the peripheral blood than HC. Newly diagnosed GCA and polymyalgia rheumatica had higher counts of classical monocytes in the peripheral blood and a reduction in the non-classical subset, but no differences concerning the intermediate subset compared to HC were found^34^.

In this study, prednisone use was associated with lower percentages of non-classical monocytes in the peripheral blood of TAK patients. In line with our findings, glucocorticoids have been shown to reduce the number of non-classical monocytes in the peripheral blood in other inflammatory conditions including GCA^34–36^. Indeed, glucocorticoid therapy induces CD16^+^ cells death in a caspase-dependent manner^37^. Despite a tendency for an overall reduction of the three subsets of monocytes in the peripheral blood after the achievement of remission, this finding did not reach statistical significance. Further analyses including a larger number of active relapsing and newly diagnosed patients with TAK would clarify the potential contributions of the longitudinal assessment of monocyte subsets as surrogate markers of disease activity and progression in the follow-up of TAK.

CCL22 was the chemokine mostly related to disease activity in this study. This chemokine seems to be relevant to TAK pathophysiology since it is produced by M2a macrophages, which play an essential role in the fibrosis process upon induction by IL-4 and IL-13^38^. The predominance of M2 macrophages infiltrating the aorta from TAK patients, observed especially in TAK patients under therapy, is in accordance with the higher production of CCL22 in TAK^24^. Persistently high serum CCL22 levels have been described in TAK patients compared to HC, and a further increase in its levels was observed even after therapy for TAK^29^. Our study, however, was the first to describe significant differences between active disease and remission regarding CCL22 serum levels in TAK.

The chemokine CCL4 is an important CCR5 ligand, which is more expressed by the intermediate than the classical subset^39^. In this study, CCL4 levels were positively correlated with total monocytes, as well as with both subtypes in TAK patients. In addition, TAK patients in remission presented lower CCL3 and CCL4 levels than HC, and that may be a reflection of immunosuppressive therapy to control disease activity. The differentiation of human monocytes into macrophages is followed by a loss of CCR2 on the surface and by an increased expression of CCR5^31^. This feature may also indicate the contribution of monocyte-derived macrophages in the peripheral depletion of circulating CCL4 and CCL3 to a lesser extent.

In contrast to results reported in previous studies^7,26,40,41^, no differences in CCL2 serum concentrations were found between TAK patients and HC. Furthermore, this chemokine was negatively correlated with the ITAS2010 score in our TAK patients. Therapy for TAK, especially prednisone use, and the remission state observed in most TAK patients evaluated in this study may have influenced this result. In addition, as systemic levels of CCL2 normalized after glucocorticoid treatment in other conditions such as GCA and systemic lupus erythematosus^34,42^, the same phenomenon may have contributed to similar CCL2 levels between TAK and HC in this study. Besides, the expression of CCL2 in the layers of the arterial wall changes in the different phases of TAK, reducing considerably after treatment^25^. Even the macrophage-infiltrate profile can modify along with TAK evolution, with a predominance of M1 macrophages in the adventitia infiltrate in the early stages of the disease followed by a remarkable increase of M2 macrophages in the media layer after treatment^24,25^. A recent study that performed a chemokine array assay in patients with TAK also did not find differences in the CCL2 average signal between patients and controls^25^.

Prednisone use was related to lower CXCL10 levels in our study. This finding indicates a possible influence of prednisone use on the production of this chemokine. Another study showed that TAK patients with active disease presented a higher CXCL10 concentration and it decreased after therapy for TAK^26^. The chemokine CXCL10 is a crucial regulator of the Th1 immune responses, and it is indeed suppressed by glucocorticoids in TAK patients^7^. Another study evaluated serum chemokines such as CXCL10, CXCL13, CCL5, and CXCL8 in TAK patients, but none showed significant differences between active disease and remission^28^. In line with these findings, Kong et al. also found no differences in the levels of CCL5, CXCL16, CXCL11, and IL‑16 between patients with active disease and those in remission^29^.

Lastly, neither non-classical monocytes nor its main chemokine CX3CL1 was related to disease status or disease activity in our TAK patients. The chemokine CX3CL1 has a crucial role in guiding non-classical monocytes to inflamed tissues as these cells have a high expression of CX3CR1^43^. In GCA, despite normal levels of CX3CL1 in patients´ plasma, a high expression of this chemokine was detected in temporal arteries^34^.

Thus, further studies approaching the expression of these chemokines, their ligands, and markers of different cell subtypes in the aorta from TAK are necessary to unravel this issue.

Limitations of this study include the assessment of patients with long-term disease duration who were under immunosuppressive therapy. We also acknowledge the low number of TAK patients who underwent longitudinal analysis as a limitation of this study.

In conclusion, we report altered counts of monocyte subsets in TAK patients for the first time with the predominance of the intermediate subset in patients over HC. Disease activity was associated with an increased number of total monocytes and the classical and intermediate subsets compared to HC, whereas CCL22 levels were surrogate markers of disease activity in TAK. Patients in remission presented lower CCL3 and CCL4 levels compared to HC. The immunosuppressive therapy had no significant impact on monocyte subsets or serum chemokines in TAK patients, except prednisone, which led to lower serum CXCL10 levels and a reduction in the percentage of non-classical monocytes in the peripheral blood.

## Methods

### Study population

We performed a cross-sectional study with a control group to assess monocytes and relevant chemokines in TAK. The inclusion criteria in the TAK group were the fulfillment of the 1990 American College of Rheumatology classification criteria for TAK^44^, age above 18 years, and written informed consent. HC were recruited among patients’ friends and university hospital workers who had age and gender distribution similar to TAK patients. This study was performed in accordance with the Declaration of Helsinki, and it was approved by the ethics committee of the Universidade Federal de São Paulo (Comitê de Ética em Pesquisa; CAAE: 56926416.9.0000.5505). All individuals included in this study gave written informed consent.

Inclusion criteria in the control group were age above 18 years and informed written consent. Exclusion criteria in both groups were pregnancy, end-stage renal disease (glomerular filtration rate < 15 mL/min/1.73 m^2^), type 1 diabetes, acute coronary syndrome in the past 6 months, previous organ transplantation, other granulomatous or rheumatic autoimmune diseases, acute or chronic infection, obesity (body mass index > 30 kg/m^2^), and history of non-skin cancer in the past 5 years. A previous history of hypertension, type 2 diabetes, and smoking was allowed in both groups.

TAK patients were evaluated for current disease activity, the extension of arterial involvement, and current therapy. Disease activity was based on a previous clinical trial assessing TAK patients and was defined by a physician with experience in TAK care (initials MFA). Active disease was considered if at least two of the five following items were present: objective systemic symptoms; subjective systemic symptoms; elevated inflammation markers; vascular signs and symptoms; ischemic symptoms^45^. At the clinical evaluation, the ITAS2010^46,47^ instrument was also applied for each patient. Chronic fatigue or elevated acute-phase reactants levels in the absence of clinical symptoms were not considered evidence of active disease. Remission was defined as the absence of any clinical symptoms directly attributable to vasculitis^48^. A relapse was defined as a reappearance of clinical disease activity after a period of remission^49^. Arterial lesions in TAK patients were assessed by vascular imaging either with contrast-enhanced computed tomography angiography, magnetic resonance angiography or Doppler ultrasound of carotid and vertebral arteries. Hata’s angiographic classification was used to describe the extension of the arterial involvement in TAK patients^50^.

Study participants underwent clinical evaluation and blood sample collection on the same day. Serum samples were stored at − 80 °C with the Protease Inhibitor Cocktail Set I—Calbiochem (Merck, Millipore, USA) until analysis. Freshly drawn peripheral blood samples in ethylenediaminetetraacetic acid (EDTA) tubes were used for the quantification of monocyte subsets.

A subgroup of TAK patients (n = 4) presenting with active disease at inclusion were reanalyzed for monocyte subsets in the peripheral blood after achieving remission. The second evaluation was performed at least 6 months after the first analysis, and patients had to be in clinical remission with a daily prednisone dose ≤ 10 mg.

### Flow cytometry

**Figure 4:**
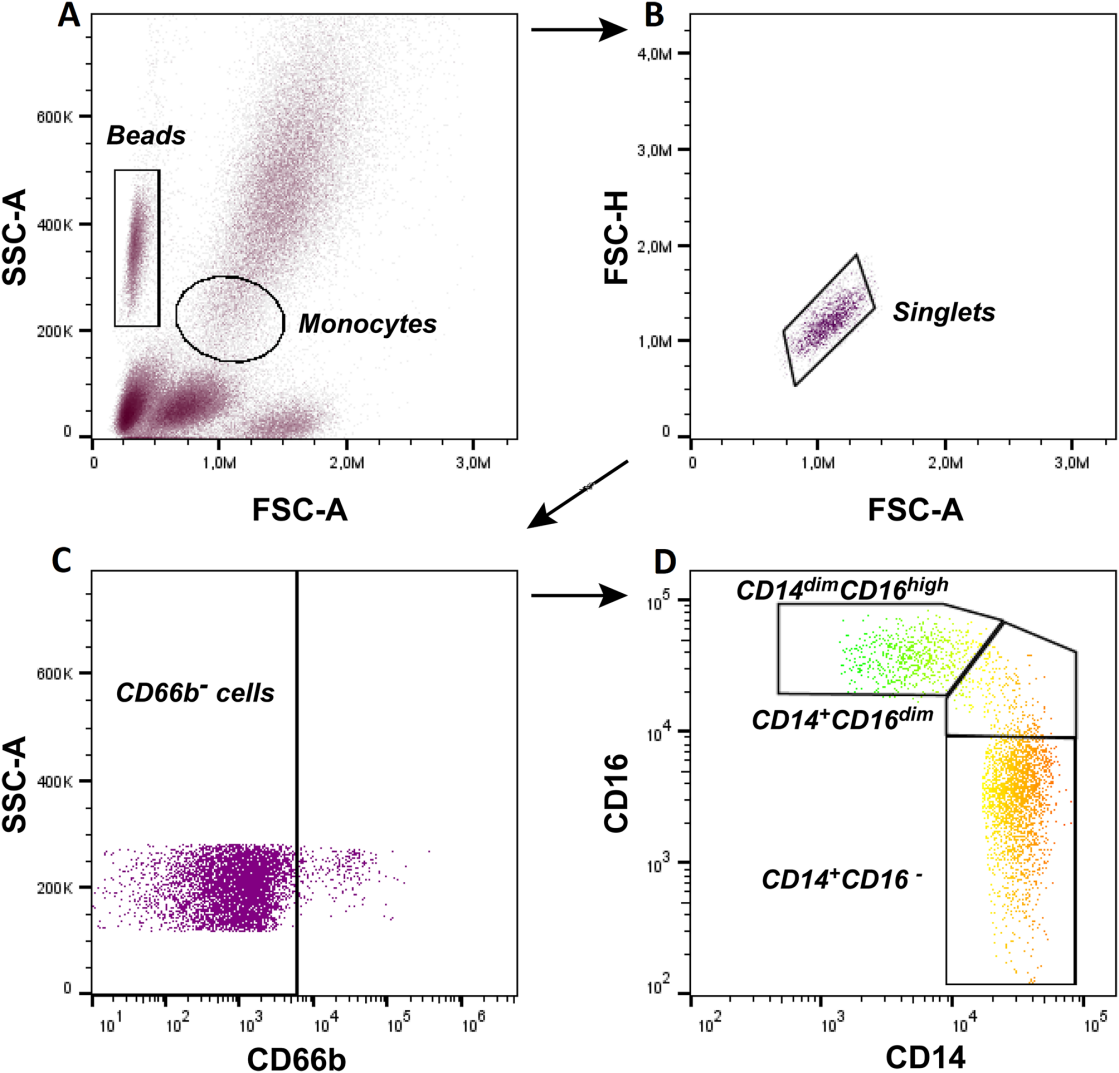
Flow cytometry analysis of monocyte subsets in the peripheral blood. Representative picture showing the gating strategy of monocytes on FCS and SSC parameters (**A**). After excluding doublets (**B**), all CD66b negative cells were selected for further analysis (**C**). Monocytes were categorized into three subtypes according to CD14 and CD16 expression as follows: classical monocytes (CD14^+^CD16^−^), intermediate monocytes (CD14^+^CD16^dim^), and non-classical monocytes (CD14^dim^CD16^high^) (**D**).

Flow cytometry experiments were performed as previously described^32^. Staining was performed in 50μL of whole blood with the following monoclonal antibodies: FITC-anti-CD14, PECy7-anti-CD16, and AF647-anti-CD66b. Samples were incubated in the dark for 15 min, and erythrocytes were lysed using the BD Fluorescence activating cell sorting (FACS) Lysing solution diluted in distilled water. After 10 min, samples were washed and resuspended in 1% bovine serum albumin (BSA) in phosphate buffered saline (PBS) and analyzed by flow cytometry using the BD Accuri C6 (BD Biosciences, New Jersey, USA). Data were collected for 10^5^ events for every sample and plotted with the Accuri C6 Software (BD Biosciences, New Jersey, USA). Fluorescence minus one (FMO) was used to correct for non-specific staining. Monocytes were gated based on forward and side scatter, and doublet cells were excluded. Contaminating granulocytes were excluded from the analysis by gating on CD66b negative cells. Monocyte subsets were then identified according to the expression of CD14 and CD16 as follows: classical (CD14^+^CD16^−^), intermediate (CD14^+^CD16^dim^), and non-classical (CD14^dim^CD16^high^) monocytes (Fig. 4). The quantification of monocytes was performed using bead-containing Trucount tubes, and results were expressed as cells ×10^6^/L and as a percentage. Flow cytometry analysis was performed using the FlowJo v8.5.3 Software for Windows (BD Life Sciences, New Jersey, USA). All reagents used were purchased from BD Biosciences, New Jersey, USA.

### Serum chemokines measurements

The multiplex technique was used to determine serum levels of CCL2, CCL3, CCL4, CCL5, CCL7, CCL22, CXCL10, and CX3CL1 (Milliplex, Merck-Millipore, USA) using the Luminex® 200 System (Luminex Corporation, USA), according to manufacturer’s instructions.

### Statistical analysis

Categorical variables were presented as absolute numbers and percentages, while continuous variables were presented as mean and standard deviation or as the median and interquartile range (IQR), accordingly. Comparisons between groups were performed using the Chi-square test or Fisher’s exact test for categorical variables and with Student’s *t*-test or the Mann–Whitney’s U test for continuous variables. For comparisons among 3 groups regarding continuous variables, one-way analysis of variance (ANOVA) or Kruskal–Wallis tests were used. Tukey’s test and Mann–Whitney’s U test were used as post hoc tests. Correlations between numeric variables were assessed using Pearson’s or Spearman’s rank correlation coefficients. The longitudinal analysis compared the paired median and IQR during active disease and remission in four TAK patients. The significance level accepted was *p* < 0.05 and *p* < 0.016 for post hoc analysis with Bonferroni´s correction in non-parametric analysis. The software IBM SPSS Statistics for Windows version 21.0 (Armonk, NY, USA) was used for statistical analysis, and graphs were built by the GraphPad Prism version 5.0 for Windows (San Diego, CA, USA).

## Supplementary Information

Supplementary Tables.

## Data Availability

The data and files used in this study are available from the corresponding authors on reasonable request.
