# Supplementary material for: Monocyte subsets and monocyte-related chemokines in Takayasu arteritis
Source: Sci Rep. 2023 Feb 6;13:2092. doi: 10.1038/s41598-023-29369-3 (PMC9902560; doi:10.1038/s41598-023-29369-3)
Supplement: Supplementary file 1 — Supplementary Tables. [file 41598_2023_29369_MOESM1_ESM.docx]

**Supplementary tables**

**Supplementary Table S1 – Serum concentration of chemokines in patients with Takayasu arteritis and healthy controls.**

| **Chemokines** | **TAK**  **(n=32)** | **Healthy controls**  **(n=30)** | ***p*** |
| --- | --- | --- | --- |
| CCL22, pg/mL | 1,121.0  (832.9-1,819.7) | 1,380.5  (992.5-1,745.0) | 0.331 |
| CX3CL1, pg/mL | 85.2  (71.2-102.6) | 98.3  (74.8-143.5) | 0.065 |
| CXCL10, pg/mL | 227.8  (138.9-362.9) | 251.1  (153.5-377.8) | 0.616 |
| CCL2, pg/mL | 696.1 ± 297.9 | 692.7 ± 372.9 | 0.968 |
| CCL3, pg/mL | 6.2  (4.9-8.1) | 9.3  (5.7-14.7) | 0.009* |
| CCL4, pg/mL | 37.4  (24.5-47.9) | 45.4  (37.7-63.7) | 0.008* |
| CCL5, pg/mL | 1,394.5  (1,157.5-1,848.0) | 1,566.5  (1,232.3-1,901.0) | 0.578 |
| CCL7, pg/mL | 38.5  (32.3-71.3) | 47.9  (35.0-59.1) | 0.284 |

n – Number of participants; TAK – Takayasu arteritis; * Flags significant results.

**Supplementary Table S2 – Correlations between monocytes subsets with ITAS2010 score and acute phase reactants.**

| **Variables** | **ITAS2010 score** | **ESR (mm/hour)** | **CRP mg/L** |
| --- | --- | --- | --- |
| Total monocytes, cells x10^6^/L | Rho: -0.264  *p* = 0.433 | Rho: -0.059  *p* = 0.761 | Rho: 0.249  *p* = 0.229 |
| Classical monocytes, cells x10^6^/L | Rho: -0.264  *p* = 0.433 | Rho: -0.066  *p* = 0.734 | Rho: 0.221  *p* = 0.288 |
| Intermediate monocytes, cells x10^6^/L | Rho: -0.127  *p* = 0.709 | Rho: -0.044  *p* = 0.822 | Rho: 0.337  *p* = 0.100 |
| Non-classical monocytes, cells x10^6^/L | Rho: 0.010  *p* = 0.977 | Rho: 0.045  *p* = 0.817 | Rho: 0.359  *p* = 0.078 |

CRP – C-reactive protein; ESR – Erythrocyte sedimentation rate; ITAS2010 – Indian Takayasu Clinical Activity Score; * - Flags significant results.

**Supplementary Table S3 – Correlations between serum chemokine levels with ITAS2010 score and acute phase reactants.**

| **Variables** | **ITAS2010 score** | **ESR (mm/hour)** | **CRP mg/L** |
| --- | --- | --- | --- |
| CX3CL1 | Rho: -0.289  *p* = 0.418 | Rho: -0.187  *p* = 0.340 | Rho: 0.148  *p* = 0.490 |
| CCL22 | Rho: -0.098  *p* = 0.775 | Rho: 0.034  *p* = 0.863 | Rho: 0.252  *p* = 0.224 |
| CXCL10 | Rho: 0.138  *p* = 0.704 | Rho: -0.175  *p* = 0.373 | Rho: -0.036  *p* = 0.867 |
| CCL2 | Rho: -0.611  *p* = 0.046* | Rho: -0.096  *p* = 0.619 | Rho: 0.143  *p* = 0.495 |
| CCL3 | Rho: 0.245  *p* = 0.469 | Rho: -0.344  *p* = 0.068 | Rho: 0.101  *p* = 0.632 |
| CCL4 | Rho: 0.274  *p* = 0.415 | Rho: -0.214  *p* = 0.265 | Rho: 0.237  *p* = 0.255 |
| CCL5 | Rho: 0.572  *p* = 0.066 | Rho: -0.014  *p* = 0.944 | Rho: 0.052  *p* = 0.804 |
| CCL7 | Rho: -0.181  *p* = 0.594 | Rho: -0.143  *p* = 0.458 | Rho: 0.295  *p* = 0.152 |

CRP – C-reactive protein; ESR – Erythrocyte sedimentation rate; ITAS2010 – Indian Takayasu Clinical Activity Score; * - Flags significant results.

**Supplementary Table S4 – Interactions between monocytes subsets and serum chemokine levels in Takayasu arteritis.**

|  | **Total monocytes** | **Classical monocytes** | **Intermediate monocytes** | **Non-classical monocytes** |
| --- | --- | --- | --- | --- |
| **CX3CL1** | Rho: 0.082  *p* = 0.661 | Rho: 0.032  *p* = 0.865 | Rho: 0.129  *p* = 0.491 | Rho: 0.139  *p* = 0.456 |
| **CCL22** | Rho: 0.079  *p* = 0.668 | Rho: 0.048  *p* = 0.796 | Rho: 0.053  *p* = 0.774 | Rho: 0.045  *p* = 0.806 |
| **CXCL10** | Rho: 0.307  *p* = 0.093 | Rho: 0.288  *p* = 0.116 | Rho: 0.272  *p* = 0.139 | Rho: 0.348  *p* = 0.055 |
| **CCL2** | Rho: 0.244  *p* = 0.179 | Rho: 0.231  *p* = 0.203 | Rho: 0.004  *p* = 0.983 | Rho: -0.033  *p* = 0.858 |
| **CCL3** | Rho: 0.250  *p* = 0.168 | Rho: 0.212  *p* = 0.244 | Rho: 0.325  *p* = 0.070 | Rho: 0.179  *p* = 0.328 |
| **CCL4** | Rho: 0.489  *p* = 0.005* | Rho: 0.448  *p* = 0.010* | Rho: 0.412  *p* = 0.019* | Rho: 0.334  *p* = 0.062 |
| **CCL5** | Rho: -0.069  *p* = 0.708 | Rho: -0.090  *p* = 0.624 | Rho: -0.137  *p* = 0.454 | Rho: 0.014  *p* = 0.940 |
| **CCL7** | Rho: 0.108  *p* = 0.556 | Rho: 0.078  *p* = 0.672 | Rho: 0.232  *p* = 0.200 | Rho: 0.360*  *p* = 0.043 |

* - Flags significant correlations.

**Supplementary Table S5 – Correlations between daily prednisone dose, monocytes in the peripheral blood and serum chemokines.**

| **Monocytes** | **Daily prednisone dose, mg** | **Serum chemokines,**  **pg/mL** | **Daily prednisone dose, mg** |
| --- | --- | --- | --- |
| **Total monocytes, cells x10^6^/L** | Rho: 0.129  *p* = 0.689 | **CX3CL1** | Rho: -0.323  *p* = 0.306 |
| **Classical monocytes, cells x10^6^/L** | Rho: 0.161  *p* = 0.616 | **CCL22** | Rho: -0.513  *p* = 0.088 |
| **Intermediate monocytes, cells x10^6^/L** | Rho: 0.280  *p* = 0.379 | **CXCL10** | Rho: -0.463  *p* = 0.130 |
| **Non-classical monocytes, cells x10^6^/L** | Rho: -0.480  *p* = 0.114 | **CCL2** | Rho: -0.014  *p* = 0.965 |
| **--** | **--** | **CCL3** | Rho: 0.244  *p* = 0.445 |
| **--** | **--** | **CCL4** | Rho: -0.011  *p* = 0.974 |
| **--** | **--** | **CCL5** | Rho: -0.190  *p* = 0.554 |
| **--** | **--** | **CCL7** | Rho: -0.492  *p* = 0.104 |
